# Tuberculin1Abstracts of an address made at the Annual Meeting of the Michigan State Veterinary Medical Association.

**Published:** 1902-07

**Authors:** H. F. Palmer

**Affiliations:** Detroit, Mich.


### TUBERCULIN.[1]

By H. F. Palmer, D.V.S.,
DETROIT, MICH.

One of the most important diagnostic agents used in the veterinary profession is tuberculin. That quiet and insidious disease, tuberculosis, which at one time was allowed to get such a hold of our bovine species, can now be recognized in its first stages, and means be taken for its eradication.

In 1882 Professor Koch announced to the scientific world that tuberculosis of whatever form was due to a specific germ, the tubercle bacillus. After eight years more of experimental work with the germ and its products, tuberculin was given to the medical profession as a remedy to be used in the treatment of tuberculosis. However, time soon showed the

[1] Abstracts of an address made at the Annual Meeting of the Michigan State Veterinary Medical Association.

useless action of this same tuberculin as a therapeutic agent and classed it as one of our diagnostic agents.

To-day tuberculin stands at the head of the list of diagnostic agents used in the veterinary profession, especially in bovine work.

Tuberculin is not an infallible guide, but from the records of those who have been the largest users of it comes the highest praise. No single agent, diagnostic or remedial, can be so implicitly relied upon as tuberculin. Dr. E. A. A. Grange, who for many years was Michigan's State Veterinarian, says of it: "I have used tuberculin in upward of one thousand instances in this State, and have not discovered a case in all these which would impeach the test." The Alabama Experiment Station says: "Out of 4068 animals in various parts of the United States, tested by various persons and with various forms of tuberculin, 1137 animals reacted and 1118 exhibited undoubted tuberculous lesions upon the post-mortem examination. In nineteen cases of those that reacted the investigator failed to find any visible tuberculous lesions. The microscope was not used, and possibly the post-mortem examinations were not as thorough and complete as they might have been." In Massachusetts the Cattle Commission have tested over 25,000 cattle, and they have found the tuberculin at fault in only one out of every 400 tested.

At an International Veterinary Congress held at Berne there was a very interesting discussion as to the use of tuberculin to diagnose the presence of tuberculosis. The weight of evidence was so much in favor of tuberculin that the vote of the congress was almost unanimous, there being but two dissidents. The French scientist, Professor Nocard, has injected tuberculin under the skin of 3000 cows, and on only two occasions did it fail, but these were in cows with the disease far advanced. M. Bang, of Denmark, produced the certificate of 45,000 cases of successful injections, with but a few failures, due to the chronic stage of tuberculosis.

The method now in vogue for manufacturing tuberculin does not essentially differ from that originally used by Koch.

Beef bouillon, to which has been added 1 to 2 per cent. of peptone, 4 to 5 per cent. of glycerin, and ½ per cent. of salt, is made slightly alkaline with sodium hydrate, and poured into sterile flasks to form a thin layer on the bottom. These flasks are then inoculated with small portions of the scale-like growth of the germ as it appears on the surface of the culture media. They are then plugged with sterilized cotton and placed in an incubator, which is kept at 37° to 38° C. In two or three weeks a slight scum appears on the culture fluid; this gradually thickens until at the end of six weeks there is quite a thick grayish-white wrinkled layer. Shortly this mass of germs breaks up into irregular pieces and falls to the bottom of the flask. These matured cultures are examined microscopically. If found pure they are poured in a shallow evaporating dish, and concentrated to one-tenth of the original volume, the result being a thick, brownish, turbid, syrupy fluid. This fluid is then filtered through unglazed porcelain, *which removes all the germs*, and we have crude tuberculin as the result. Upon adding 60 per cent. of alcohol to this crude tuberculin a precipitate is thrown down, which, after being washed several times with dilute alcohol and dried in vacuo, constitutes the purified tuberculin of Koch.

Tuberculin is placed on the market as a syrupy liquid in concentrated form, to be used with some diluent, as $\frac{1}{2}$ per cent. carbolic acid, distilled water, or normal salt solution.

Dose: 0.4 of a cubic centimetre (6 drops) with an indefinite quantity of the diluent.

Injection: Tuberculin should be given as a regular hypodermic injection. The usual precaution of sterilizing the site of injection and using the sterile syringe and needle should not be neglected. Dilute the tuberculin and inject just beneath the skin at some convenient point along neck or fore-shoulder region.

Reaction: The temperature of the animal should be observed several hours before injection in order to ascertain the normal temperature of each individual. Do not inject an animal with an irregular temperature or one that has an abnormal temperature, either high or low, for in such cases it cannot be ascertained whether it is the action of the tuberculin or a previous disordered condition (not tubercular) that causes rise of temperature. Ten hours after injection the temperature of the animal should be taken every two hours. Some rely on the temperatures taken at the twelfth, fifteenth and eighteenth hours after injection for a reaction, but it is the best noted by taking six or seven temperatures some two hours apart. A rise of two degrees or over from the normal temperature previous to injection constitutes a reaction.

Just why we get a rise of temperature after the injection of tuberculin in tuberculous animals is a question of much controversy. Some explain it by saying that the slightly diseased system contains a certain amount of the chemical poison analogous to the toxin which we inject. To this small amount of toxin the system is accustomed, and the reaction it causes is not perceptible. But when in addition to this we introduce into the system a small quantity of the poisonous product of the bacilli used for the test, the increased quantity acts on the diseased tissues and nerve centres alike, and so fever and other constitutional symptoms result.

Frequently the use of other toxins in immunizing animals for the production of serums has given us similar results. A supposedly healthy horse may respond to the injection of even one drop of toxin by producing a rise of 4° in temperature, and remaining at this point for three days. Can we not from this infer that an animal even slightly diseased will the more readily react to this small dose of toxin?

During the testing period the animal should be kept under as uniform conditions as possible, as a change in conditions will often cause a change of a degree in the temperature. It is advisable to keep the animal closely housed in order that the above conditions can be met.

To a healthy animal there is no reaction, neither fever nor constitutional. The milk of a milch cow is unaffected, and can be used as food the same as though not injected. Besides the rise of temperature in a reacting animal there will often be noticed a large swelling at point of injection, malaise, anorexia, which usually passes off at the end of twenty-four hours.

Below we give the record of two animals under test, each of which was injected at 9 P.M. No. 1, showing no reaction, was pronounced healthy. No. 2 gave a rise of $3\frac{2}{5}$°, which is a distinct reaction, and the animal was pronounced tuberculous.

*Before Injection.*

| | 9 | 2 | 3 | 6 | 9 |
|---|---|---|---|---|---|
| No. 1. | 101 | 101 | 101 | 101 | 102 |
| " 2. | 102 | 102 | 102 | 102 | 102 |

*After Injection.*

| | 6 | 8 | 10 | 12 | 2 | 4 | 6 |
|---|---|---|---|---|---|---|---|
| No. 1. | 102 | 101 | 102 | 101 | 101 | 101 | 101 |
| " 2. | 105 | 106 | 105 | 103 | 103 | 103 | 103 |

Criticisms have arisen regarding the use of tuberculin, but the following facts will prove these objections unfounded:

1. Healthy animals may be made tuberculous by injections of tuberculin. This is impossible, as the germs are not present in tuberculin. The toxic product (tuberculin) is rendered sterile by high temperature or by passing through a porcelain filter.

2. Animals in an advanced stage of tuberculosis do not react. This is often the case, but tuberculosis can usually be detected in such animals by a physical examination.

3. After one reaction from the use of tuberculin the animal's ability to react is lost. In a measure this is true, and we would advise not to inject unless at least one month has elapsed since the last injection. Some unscrupulous dealers who desire to barter a tuberculous animal will try to disguise a tuberculous reaction by giving the animal a dose of tuberculin a few days previous to time of regular test.

4. Some think that tuberculin has the power of arousing latent tuberculous foci, and producing a much wider spread of the disease. To this objection we would refer to the experience of Nocard, who from more than three thousand tuberculin injections has only in three cases, and those far advanced in the disease, observed aggravation of tuberculosis by tuberculin injections.

### Mallein.

Another important diagnostic agent used in the veterinary profession is mallein. Although not as infallible as tuberculin, still time and experience give it a very important place. This agent is used to detect glanders in all equines.

Beef bouillon is prepared in the usual manner and put into sterile flasks which are planted with a pure culture of bacillus mallei. These flasks are put in an incubator and kept at a temperature of 37° to 38° C. In a short time a growth is noticed, and at the end of four to six weeks a thick scum has formed. The germs in growing elaborate a chemical poison-toxin, which is the crude mallein. The flask is now taken from the incubator, heated at 212° for one hour, which kills all the germs. The preparation is then filtered, first through paper, and finally through a Berkfeldt filter, which removes all germs. This filtrate is again heated and concentrated to one-tenth of its original volume.

Dose: 1 c.c. of the concentrated filtrate with an indefinite quantity of any diluent, as ½ per cent. carbolic acid, distilled water, or normal salt solution.

Injection: Dilute the mallein and inject just beneath the skin of the neck or fore-shoulder region. The site of injection should be sterilized, and only a sterile needle and syringe employed.

Reaction: Like tuberculin, the reaction is best noted if several temperatures are taken at intervals previous to injection, to ascertain the normal temperature of each animal. If the bacillus of glanders is present in the system, some ten hours after injection the temperature will begin to rise, and keep on the ascending scale for six or eight hours, then gradually descending to normal. A rise of two degrees or over from normal constitutes a reaction. Besides the temperature reaction there will usually be local manifestations. A large, painful swelling will appear at point of injection. The animal will appear weak, trembling and dejected-looking. These abnormal symptoms all subside in a few days.

The reason why we get a reaction from a horse with glanders and no reaction from any other disease is probably analogous to the tuberculous reaction following the injection of tuberculin. Dr. J. M. Wright, Assistant State Veterinarian, of Illinois, injected mallein into horses known to be affected with other diseases than glanders, and not in a single instance was there a reaction simulating the mallein reaction. He used six horses with well-marked generalized melanosis, thirteen cases of pleurisy, sixteen cases of pneumonia, three cases of pleuropneumonia, twenty-four cases of influenza, three cases of purpura hemorrhagica, fourteen cases of lymphangitis, two of which were in a state of suppuration. He says of them: "None of these animals showed the slightest degree of reaction, either by causing an attenuation of temperature or systemic disturbance." Mallein is a guide to detect bacillus mallei, and that alone.

Below we give the temperature reaction in two cases. Each animal was injected at 9 P.M. No. 1 had no appreciable rise of temperature, consequently was pronounced healthy, while No. 2 had a rise of 3⅗° in temperature, and so was pronounced glanderous.

*Before Injection.*

| | 9 | 12 | 3 | 6 | 9 |
|---|---|---|---|---|---|
| No. 1. | 100 | 100 | 100.2 | 100.1 | 100.2 |
| " 2. | 100.2 | 100 | 100.1 | 100 | 100 |

*After Injection.*

| | 6 | 8 | 10 | 12 | 2 | 4 | 6 |
|---|---|---|---|---|---|---|---|
| No. 1. | 100 | 100 | 100.3 | 100.1 | 100.2 | 100.3 | 100.2 |
| " 2. | 100 2 | 101.2 | 101 3 | 104 | 100.3 | 102.1 | 101.3 |

The same criticisms are urged against the use of mallein as against tuberculin, saying it is not infallible, that it will produce the disease, and cause a chronic case of glanders to become acute. To these criticisms we would say that mallein when properly prepared will no more induce the disease glanders than as though so much distilled water had been injected. The mallein is free from germs, having been rendered sterile either by heat or by filtration through unglazed porcelain. Even though a mallein injection does render a chronic case of glanders acute, is it not better to hasten the termination of this fatal disease than to keep an animal that is continually giving off the deadly germs?

